# A TLR7 agonist enhances the antitumor efficacy of obinutuzumab in murine lymphoma models via NK cells and CD4 T cells

**DOI:** 10.1038/leu.2016.352

**Published:** 2017-01-03

**Authors:** E J Cheadle, G Lipowska-Bhalla, S J Dovedi, E Fagnano, C Klein, J Honeychurch, T M Illidge

**Affiliations:** 1 Targeted Therapy Group, Division of Molecular and Clinical Cancer Sciences, University of Manchester, Christie Hospital, Manchester Academic Health Sciences Centre, Manchester, UK; 2 Roche Pharmaceutical Research & Early Development, Roche Innovation Center Zurich, Zurich, Switzerland

## Abstract

Anti-CD20 monoclonal antibodies (mAb) such as rituximab have been proven to be highly effective at improving outcome in B-cell malignancies. However, many patients ultimately relapse and become refractory to treatment. The glycoengineered anti-CD20 mAb obinutuzumab was developed to induce enhanced antibody-dependent cellular cytotoxicity, antibody-dependent phagocytosis and direct cell death and was shown to lead to improved outcomes in a randomized study in B-CLL. We hypothesized that immune stimulation through Toll-like receptor 7 (TLR7) agonism in combination with obinutuzumab would further enhance lymphoma clearance and the generation of long-term antitumor immune responses. Here we demonstrate, in syngeneic human CD20 (hCD20)-expressing models of lymphoma, that systemic administration of a TLR7 agonist (R848) increases responses when administered in combination with obinutuzumab and protects against disease recurrence. Depletion studies demonstrate that primary antitumor activity is dependent on both NK cells and CD4^+^ T cells but not on CD8^+^ T cells. However, both CD4^+^ and CD8^+^ T cells appear necessary for the generation of protective immunological memory. Importantly, increased tumor-free survival post obinutuzumab and R848 combination therapy was seen in hCD20 transgenic mice, which express hCD20 on normal B cells. These findings provide a rationale for clinical testing of obinutuzumab in combination with systemically administered TLR7 agonists to further improve outcome.

## Introduction

Non-hodgkin lymphoma and chronic lymphocytic leukemia account for ~9% of all new cancers diagnosed in the United States annually and continue to represent a significant therapeutic challenge.^1^ The anti-CD20 monoclonal antibody (mAb) rituximab has significantly improved survival^2, 3^ but many patients ultimately relapse, necessitating the development of novel therapies and improved anti-CD20 mAbs. The glycoengineered anti-CD20 mAb obinutuzumab was developed to have enhanced antibody-dependent cellular cytotoxicity (ADCC)^4^ and ADCP (antibody-dependent phagocytosis)^5^ owing to enhanced FcγRIII-binding affinity and induces profound direct programmed cell death.^6^ A number of *in vitro* and pre-clinical xenograft studies demonstrated the superiority of obinutuzumab over rituximab,^7^ which was confirmed in a phase III trial in chronic lymphocytic leukemia, leading to its licensing by the FDA^8^ and in combination with bendamustine for the treatment of rituximab refractory/relapsed follicular lymphoma.^9^

Evidence suggests that adaptive immunity may have a role in durable responses seen after anti-CD20 mAb therapy with pre-treatment T-cell levels linked to clinical outcome post rituximab^10^ and the presence of idiotype-specific T cells post treatment.^11^ Furthermore, we have demonstrated that obinutuzumab induces the release of damage-associated molecular pattern molecules, which can prime dendritic cell maturation and T-cell activation.^12^ Recent data have demonstrated the importance of the tumor microenvironment in regulating T-cell responses, which has led to intense interest in manipulating the balance between positive immune-stimulatory signals and negative regulatory signals with immuno-modulatory agents.^13^

Toll-like receptors (TLR) are expressed on immune cells which, upon engagement by damage-associated molecular pattern molecules and pathogen-associated molecular patterns, trigger a cascade of signaling pathways, leading to production of pro-inflammatory cytokines, polarization of T-cell responses and activation of antigen presenting cells. TLR7 is an endosomally located receptor whose natural ligand is viral uridine- and guanosine-rich single-stranded RNA. Synthetic agonists of TLR7/8 have been shown to activate plasmacytoid and myeloid dendritic cells, stimulate production of type I interferons and stimulate strong T_H_-1 immunity and CD8^+^ T-cell responses.^14, 15^ The only TLR7/8 agonist licensed to date (Imiquimod) is currently administered as a topical treatment for basal cell carcinoma and other dermatological malignancies. Recently, topical administration of resiquimod (R848) was shown to induce regression of both treated and non-treated cutaneous T-cell lymphoma lesions, suggesting the induction of adaptive immunity, which was further evidenced by the expansion of benign T-cell clones and effector function.^16^ We have previously shown that systemic administration of TLR7 agonist (R848) in combination with radiation can prime CD8^+^ T-cell responses, which mediate antitumor activity in murine lymphoma models.^17^ A number of novel TLR7/8 agonists are currently in pre-clinical development and clinical testing (NCT02556463). Therefore, we chose to use R848, which binds selectively to mouse TLR7, to develop a syngeneic murine lymphoma model to investigate whether TLR7 agonism can enhance the efficacy of anti-CD20 antibodies by priming of T-cell responses. We demonstrate that R848 can enhance the therapeutic efficacy of obinutuzumab, leading to long-term survival and antitumor immunity through an NK and CD4^+^ T-cell-dependent mechanism, providing proof of principle for translation to the clinic.

## Materials and methods

### Antibodies and reagents

obinutuzumab, obinutuzumab m2a (Obz m2a, humanized Fab region from obinutuzumab with the human IgG_1_ Fc region replaced with a glycoengineered murine IgG2a Fc region) and rituximab m2a (rituximab with murine IgG2a Fc constant region) were produced by transient expression at Roche Innovation Centre Zurich. All other antibodies were obtained from eBioscience (Hatfield, UK) and media from Invitrogen (Paisley, UK) unless stated otherwise.

### Human samples

Ethical approval for B-chronic lymphocytic leukemia (B-CLL) samples was obtained from the Manchester Cancer Research Center Biobank ethics committee and for healthy donor peripheral blood mononuclear cells from the South Manchester Ethics committee in accordance with the declaration of Helsinki. Peripheral blood mononuclear cells were isolated from patients at the Christie Hospital NHS trust (Manchester, UK) after informed consent.

### Mice and cell lines

C57Bl/6 mice were obtained from Envigo (Loughborough, UK) and NOD.Cg-Prkdc^scid^ Il2rg^tm1Wjl^/SzJ (NOD *scid* gamma) mice from JAX labs and bred in-house at the Cancer Research UK Manchester Institute (CRUK-MI), UK. Human CD20 (hCD20) transgenic mice^18^ were a gift from Professor M Cragg (University of Southampton, UK) and Professor M Shlomchik (Yale University, USA) and bred in-house at the CRUK-MI. All *in vivo* studies were carried out under the auspices of the 1986 ASPA act and EU directive 2010/63 under UKCCCR guidelines, approved by a local ethical committee and performed under a UK Home Office license. Mice were housed in specific pathogen-free facilities. For therapy studies group sizes were 5–7 animals and experiments were repeated at least once. Mice were culled when they developed palpable tumor masses or CNS disease (onset of hind leg paralysis).

The EL4 (TIB-39) and Raji (CCL-86) lymphoma cell lines were obtained from ATCC (LGC Standards, Teddington, UK) and maintained in RPMI 1640 supplemented with 10% FCS, 2 mM L-glutamine, 25 mM Hepes (Sigma-Aldrich, Poole, UK) and 50 nM 2-mercaptoethanol. Cells were routinely screened to confirm absence of mycoplasma.

### Generation of EL4-expressing hCD20

EL4hCD20 cells were generated as described in Supplementary Methods (Supplementary Figure 1).

### Tumor therapy

Mice were inoculated intravenously with 5 × 10^5^ EL4-hCD20 via the tail vein. In total, 50 μg obinutuzumab m2a or saline was administered intra-peritoneal (i.p.) on day 1, 4, 7, 11 and 14 (C57Bl/6) or day 7, 11, 14, 18, 21 (hCD20Tg). R848 (Enzo Life Sciences, Exeter, UK) was administered intravenously at 3 mg/kg on day 1, 7, 14 and 21 or day 7, 14, 21 and 28. For cellular depletion experiments mice received αCD8 mAb, clone YTS169.4 (500 μg i.p. on day 1, 4, 7 and 11; BioXcell, 2B Scientific, Upper Heyford, UK; #BE0117); αCD4 mAb, clone GK1.5 (250 μg i.p on day 1 and 8; BioXcell, #BE0003-1); αAsialo-GM1 (50 μl i.p. on day 1, 4, 7 & 11; Alpha Laboratories, Eastleigh, UK, #986-10001); α NK1.1 mAb, clone PK136 (100 μg i.p. on day 1, 4, 7 and 11; eBioscience, #16-5941); or combinations thereof. Cellular depletion was confirmed as detailed in Supplementary Methods.

### Detection of cytokine secreting T cells from treated mice

Splenocytes were co-cultured with irradiated EL4hCD20 tumor cells (30 Gy) at a ratio of 3.5:1 for 5 days in 100 IU/ml human IL-2 (Proleukin, Prometheus, San Diego, CA, USA) and re-stimulated for a further 17 h with irradiated EL4hCD20 tumor cells at a 1:1 ratio in 100 IU/ml IL-2 and Brefeldin-A. Cells were stained as detailed in Supplementary Methods.

### Assessment of NK cell activation in treated mice

Splenocytes were cultured for 4 h in the presence of Brefeldin A or monensin and anti-CD107a-PE (eBioscience, #12-1071) at 10^7^/ml in 96-well plates pre-coated with 10 μg/ml obinutuzumab m2a overnight at 4 °C in borate buffer where indicated. Cells were stained for interferon (IFN) γ as detailed in Supplementary Methods.

### ADCC and ADCP

ADCC and ADCP assays were performed as described in the Supplementary Methods.

### Statistical methods

All statistical analysis was undertaken using GraphPad PRISM software as detailed in Supplementary Methods. *P*-values <0.05 were deemed significant.

## Results

### Systemic administration of a TLR7 agonist activates immune cells *in vivo* and enhances obinutuzumab-mediated antitumor effector mechanisms *in vitro*

**Figure 1:**
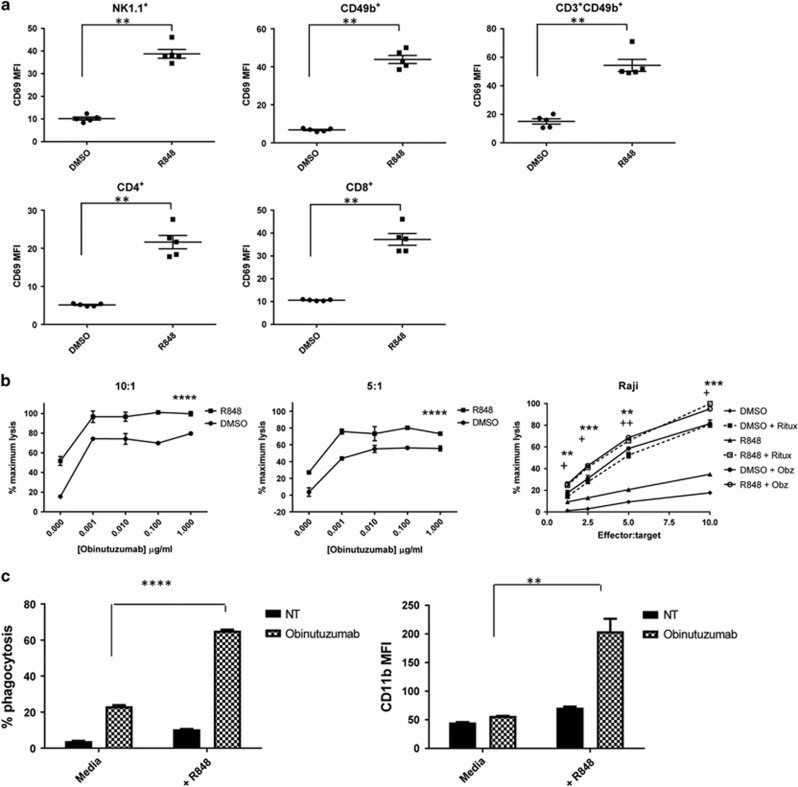
Systemic administration of the TLR7 agonist R848 activates NK cells and T cells *in vivo* and enhances anti-CD20 mAb effector mechanisms *in vitro*. (**a**) C57Bl/6 mice (*n*=5) received an iv injection of R848 (3 mg/kg) or DMSO control via the tail vein. After 20 h mice were culled and splenocytes analyzed for expression of NK1.1, CD49b, CD3, CD4, CD8 and CD69 by flow cytometry. Median fluorescent intensity (MFI) CD69 expression is shown. **P<0.01, Mann–Whitney test. (**b**) Human PBMC from healthy donors were activated for 20 h with 30 μM R848 (▪) or DMSO (•) equivalent. NK cells were isolated and cultured with 5000 calcein-AM labeled Raji cells for 2 h in the presence or absence of varying concentrations of obinutuzumab at a 10:1 or 5:1 effector: target ratio in duplicate wells (left hand and middle panel). Alternatively, NK cells were cultured at decreasing effector:target ratios with Raji cells in the presence or absence of 1 μg/ml obinutuzumab or rituximab (right hand panel) in triplicate wells. % ADCC was measured by measurement of calcein release into the supernatant compared with maximum lysis in 4% Triton. Data are presented as mean ± s.e.m and are representative of 2–3 independent experiments ****P<0.0001, two-way ANOVA (left hand and middle panel). ***P<0.001, **P<0.01, *P<0.05 unpaired students *t*-test (* rituximab, + obinutuzumab, right-hand panel). (**c**) Neutrophils were isolated from the bone marrow of C57Bl/6 mice and cultured for 2 h with or without 10 μM R848. Neutrophils were then cultured for a further 20 h with PKH26 labeled EL4hCD20 cells in the presence or absence (no treatment, NT) of obinutuzumab before labeling with anti-CD11b and analysis by flow cytometry. % phagocytosis (calculated as the percentage of CD11b^+^ cells that had taken up PKH26^+^ cells) and CD11b median fluorescence intensity is shown. Data are mean plus s.e.m of triplicate wells and are representative of two independent experiments **P<0.01, ****P<0.0001, unpaired student *t*-test.

Systemic administration of the TLR7 agonist R848 led to the activation of NK (NK1.1^+^/CD49b^+^), NKT (CD3^+^, CD49b^+^), CD4^+^ and CD8^+^ cells as evidenced by upregulation of CD69 median fluorescent intensity (*P*<0.01) (Figure 1a) and percentage CD69^+^ cells (Supplementary Figures 2a and b).

Given the ability of R848 to activate NK cells *in vivo,* we next determined whether pre-treatment of peripheral blood mononuclear cells with R848 enhanced NK cell-mediated anti-CD20mAb-specific ADCC. A statistically significant increase in ADCC was seen when R848 pre-treated NK cells were cultured with obinutuzumab or rituximab opsonized Raji cells (Figure 1b). Furthermore, R848 pre-treatment of neutrophils led to significantly enhanced obinutuzumab-mediated phagocytosis of EL4hCD20 cells and neutrophil activation (as evidenced by upregulation of CD11b) compared with control neutrophils (Figure 1c, Supplementary Figure 2c). These data show that systemic administration of the TLR7 agonist R848 can activate immune effector cells and enhance anti-CD20 mAb effector mechanisms, which led us to determine whether combining obinutuzumab with R848 would significantly enhance efficacy *in vivo*.

### Combining R848 with anti-CD20 antibodies significantly enhances the survival of C57Bl/6 mice bearing hCD20^+^ lymphoma

To establish whether systemic administration of a TLR7 agonist could improve obinutuzumab efficacy we developed a syngeneic model of lymphoma in immune-competent mice. This enabled us to investigate whether any enhanced efficacy was through priming of antitumor immunity as therapies which lead to prevention of relapse are most likely to show durable clinical outcomes.

**Figure 2:**
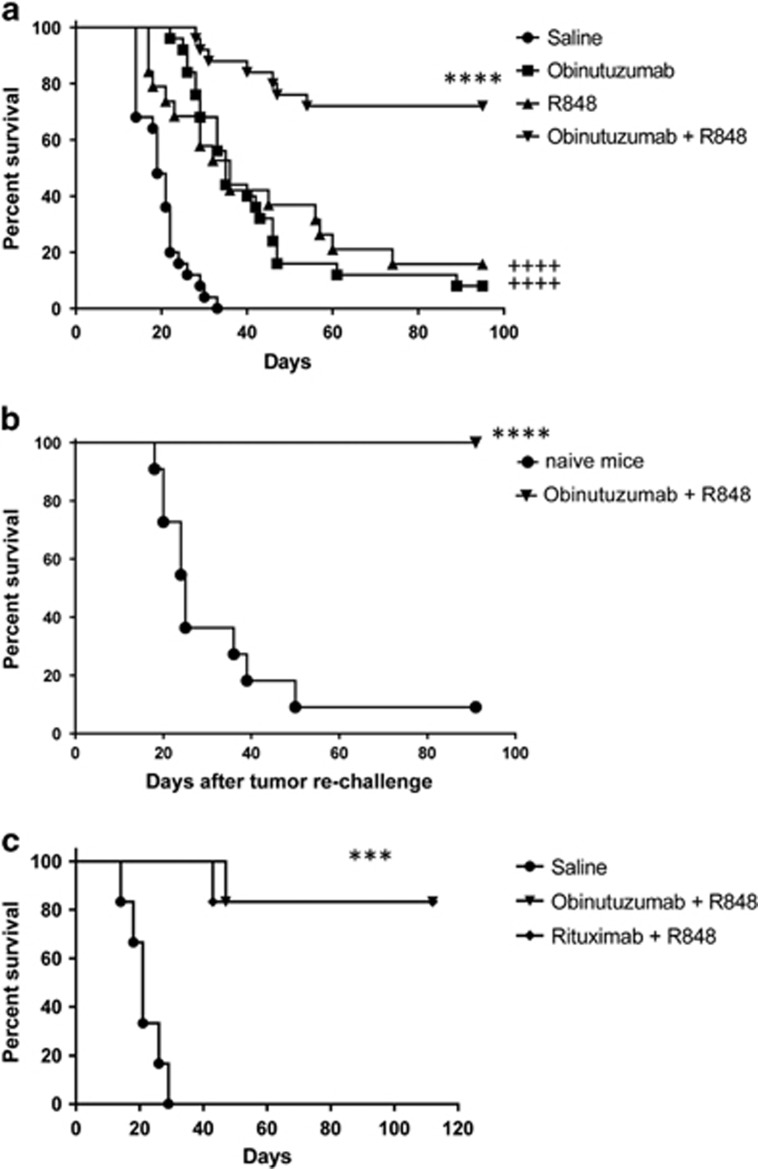
The TLR7 agonist R848 significantly enhances the therapeutic efficacy of the anti-CD20 mAb obinutuzumab and long-term survivors are protected from tumor rechallenge. (**a**) C57Bl/6 mice were injected with 5 × 10^5^ EL4hCD20 cells i.v. via the tail vein on day 0. Mice received i.p. injections on day 1, 4, 7, 11 and 14 of saline (•) or 50 μg obinutuzumab m2a (▪,▾) and i.v. injections of 3 mg/kg R848 via the tail vein on day 1, 7, 14 and 21 (▴,▾). Kaplan–Meier survival curve is shown of data pooled from four independent experiments with 5–7 mice per group (saline *n*=25, obinutuzumab *n*=25, R848 *n*=19, obinutuzumab+R848 *n*=25). ****P<0.0001, compared with obinutuzumab (log-rank; Mantel–Cox test), ^++++^*P*<0.0001, compared with saline (log-rank; Mantel–Cox test). (**b**). Long-term survivors or naive C57Bl/6 controls were rechallenged with 10^5^ EL4hCD20 i.v. at day 104 or 125. Kaplan–Meier survival curve is shown (*n*=11). Day 0 represents the day of tumor rechallenge. Data are pooled from two independent experiments. ****P<0.0001, compared with naive control mice (log-rank; Mantel–Cox test). (**c**) C57Bl/6 mice were injected with 5 × 10^5^ EL4hCD20 cells i.v. via the tail vein on day 0. Mice received i.p. injections on day 1, 4, 7, 11 and 14 of saline (•), 50 μg obinutuzumab m2a and i.v. injections of 3 mg/kg R848 via the tail vein on day 1, 7, 14 and 21 (▾) or 50 μg rituximab m2a and i.v. injections of 3 mg/kg R848 via the tail vein on day 1, 7, 14 and 21 (♦). Kaplan–Meier survival curve is shown (*n*=6 per group). *** *P*<0.001, compared with saline control mice (log-rank; Mantel–Cox test).

C57Bl/6 mice bearing systemic EL4hCD20^+^ cells received obinutuzumab modified to express the murine glycoengineered IgG_2a_ Fc region (m2a) starting 1 day after tumor inoculation and systemic R848 once weekly for 4 weeks. Whereas obinutuzumab and R848 mono-therapy significantly increased survival compared with controls (Figure 2a, *P*<0.0001) only 8–15% were long-term survivors (>90 days, LTS). However, combining obinutuzumab with R848 led to a significant increase in survival compared with either mono-therapy (*P*<0.0001) with ~70 percent of mice remaining tumor-free out to 95 days. Importantly, LTS that had received the combination therapy were protected from tumor rechallenge (Figure 2b, *P*<0.0001). Furthermore, splenocytes from LTS had a significantly greater frequency of IFNγ producing CD4^+^ T cells compared with naive control mice when cultured with irradiated EL4hCD20 cells (Supplementary Figure 3, *P*=0.048).

Although obinutuzumab has been shown to be superior to rituximab in Phase III clinical trials, rituximab remains the most commonly used anti-CD20 antibody in the clinic. We therefore compared obinutuzumab with rituximab in combination with R848 and found that both combinations were equally as effective with 80% LTS (Figure 2c, *P*=0.0005 versus saline, *P*=0.94 obinutuzumab plus R848 versus rituximab plus R848). Given that rituximab is non-glycoengineered this suggests that the glycoengineering of obinutuzumab was not important in this model.

### Obinutuzumab and R848 combination therapy efficacy is dependent on CD4 and NK cells

In order to improve clinical outcome for patients receiving anti-CD20 mAb the generation of long-term immunological memory is key to preventing tumor relapse. As obinutuzumab plus R848 combination treated LTS were protected from tumor rechallenge this suggests priming of adaptive immunity. We therefore investigated the role that T cells and NK cells had during therapy.

**Figure 3:**
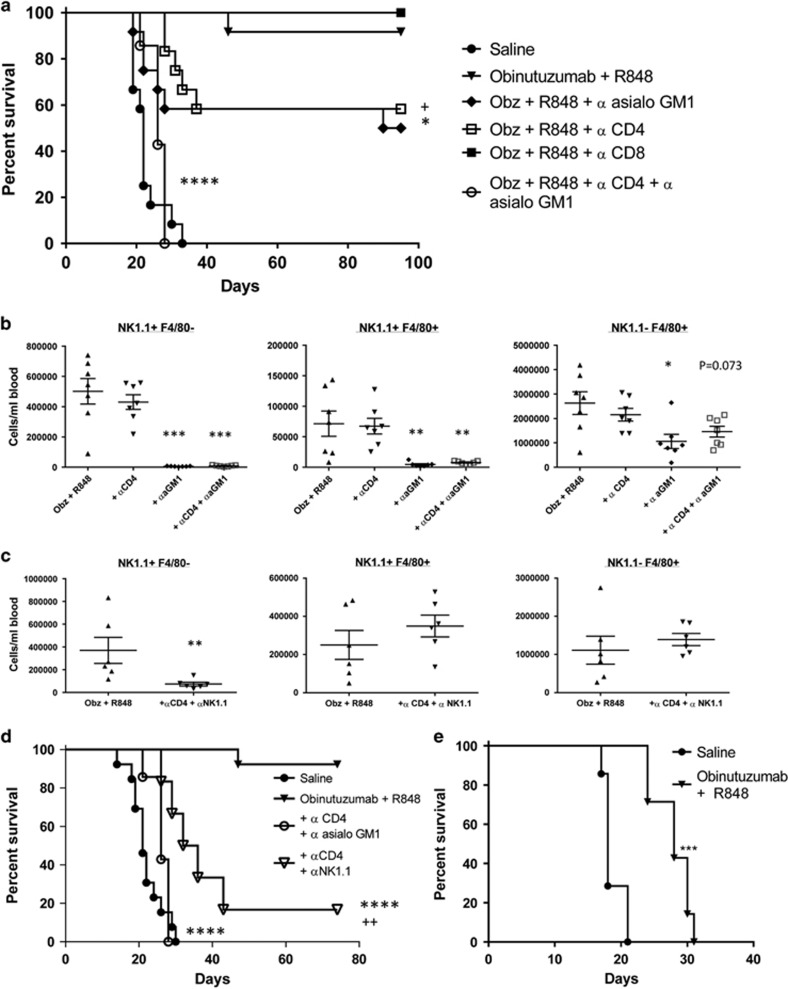
Therapeutic efficacy of obinutuzumab and R848 combination therapy is dependent on NK cells and CD4 T cells. (**a**) C57Bl/6 mice were injected with 5 × 10^5^ EL4hCD20 cells i.v. via the tail vein on day 0. Depletion antibodies were given i.p. 4 h prior to mAb on day 1, 4, 7, 11 (500 μg anti-CD8, 50 μl anti-asialo-GM1) or day 1 and 8 (250 μg anti-CD4). Mice received saline i.p. on day 1, 4, 7, 11 and 14 (•) or 50 μg obinutuzumab m2a and i.v injections of 3 mg/kg R848 via the tail vein on day 1, 7, 14 and 21 (all remaining groups). Kaplan–Meier survival curve is shown of data pooled from one to two independent experiments (saline (•, *n*=12), obinutuzumab+R848 (▾, *n*=12), obinutuzumab+R848+αCD8 (▪, *n*=5), obinutuzumab+R848+αCD4 (□, *n*=12), obinutuzumab+R848+α asialo-GM1 (♦, *n*=12), obinutuzumab+R848+αCD4+α asialo-GM1 (○, *n*=7)). *P<0.05, ****P<0.0001 compared with obinutuzumab+R848 (log-rank; Mantel–Cox test). +*P*<0.05 compared with obinutuzumab+R848 (Gehan–Breslow–Wilcoxon test). (**b**) Tail bleeds were taken on day 4 from mice treated as above and weighed and the number of NK1.1^+^ F4/80^+^ cells was analyzed by flow cytometry. The number of cells/ml of blood was calculated using CountBright beads. (α aGM1=α asialo GM1). The gating strategy is shown in Supplementary Figure 6a. (**c**) C57Bl/6 mice were treated as described in **a** with obinutuzumab+R848 or obinutuzumab+R848+αCD4+αNK1.1. Tail bleeds were taken on day 4 and weighed and the number of NK1.1^+^ F4/80^+^ cells was analyzed by flow cytometry. The number of cells/ml of blood was calculated using CountBright beads. The gating strategy is shown in Supplementary Figure 6b. (**d**) C57Bl/6 mice were injected with 5 × 10^5^ EL4hCD20 cells i.v. via the tail vein on day 0. Depletion antibodies were given i.p. 4 h prior to mAb on day 1, 4, 7, 11 (100 μg αNK1.1, 50 μl α asialo-GM1) or day 1 and 8 (250 μg αCD4). Mice received saline i.p. on day 1, 4, 7, 11 and 14 or 50 μg obinutuzumab m2a and i.v injections of 3 mg/kg R848 via the tail vein on day 1, 7, 14 and 21. Kaplan–Meier survival curve is shown of data pooled from one or two independent experiments (saline (•) *n*=12, obinutuzumab+R848 (▾) *n*=12, obinutuzumab+R848+αCD4+α asialo-GM1 (○) *n*=7, obinutuzumab+R848+αCD4+αNK1.1 (∇) *n*=6). ****P<0.0001 compared with obinutuzumab+R848 (log-rank; Mantel–Cox test) ++*P*<0.01 compared with saline. (**e**) NOD *scid* gamma (NSG) mice were injected with 5 × 10^5^ EL4hCD20 cells i.v. via the tail vein on day 0. Mice received saline (•) or 50 μg obinutuzumab m2a i.p. on day 1, 4, 7, 11 and 14 and i.v injections of 3 mg/kg R848 via the tail vein on day 1, 7, 14 and 21 (▾). Kaplan–Meier survival curve is shown (*n*=7 per group). ***P<0.001, compared with saline control mice (log-rank; Mantel–Cox test).

CD4^+^, CD8^+^ T cells and NK cells were depleted over the course of therapy (confirmed by tail bleed analysis at day 4, Supplementary Figure 4a). Depletion of either CD4^+^ T cells or NK cells led to a reduction in LTS in the combination therapy (*P*=0.0506 (CD4) and *P*=0.02 (NK cells)) but CD8^+^ T cells were not required for therapy (Figure 3a). Moreover, concomitant depletion of both CD4^+^ T cells and NK cells led to complete abrogation of therapeutic efficacy compared with obinutuzumab plus R848 (*P*<0.0001). These results suggest that obinutuzumab plus R848 combination therapy is mediated by both NK cells and CD4 T cells. Furthermore, a significant increase in the NK cell population (NK1.1^+^ or CD11b^lo^) was seen in the peripheral blood on day 4 of therapy following combination therapy (Supplementary Figure 5). As anti-asialo GM-1 also decreased circulating F4/80^+^ cells in the blood, which may be monocytic in origin, (Figure 3b) we repeated studies using a depleting antibody to NK1.1. In keeping with our previous observation, depletion of CD4^+^ T cells and NK1.1^+^ cells using this approach significantly reduced the efficacy of obinutuzumab plus R848 (Figure 3d, *P*<0.0001) but no depletion of F4/80^+^ cells was seen (Figure 3c). Although a small but significant increase in survival over controls was seen (*P*=0.001), we hypothesize this was due to the reduced efficacy of NK1.1-mediated depletion upon the NK1.1^Hi^ F4/80^-^ population compared with anti-asialo GM-1 depletion (*P*=0.0012, Supplementary Figure 4b).

To further investigate a role for monocytes or macrophages in mediating therapeutic efficacy we treated EL4hCD20-bearing NOD *scid* gamma mice, which lack T cells and B cells and have a very low number of non-functioning NK cells, with obinutuzumab plus R848. Although no LTS were seen (Figure 3e) there was around a 10 day increase in survival over controls (*P*<0.001), suggesting that macrophage/monocyte effector mechanisms may have a role in short-term tumor cell depletion. However, taken with the depletion studies, we believe these data exclude a role for monocytes/macrophages in mediating long-term survival post-obinutuzumab and R848 combination therapy, which appears to be mediated solely by NK cells and CD4^+^ T cells.

### R848 activates NK cells *in vivo* to enable engagement of NK cell function by obinutuzumab

**Figure 4:**
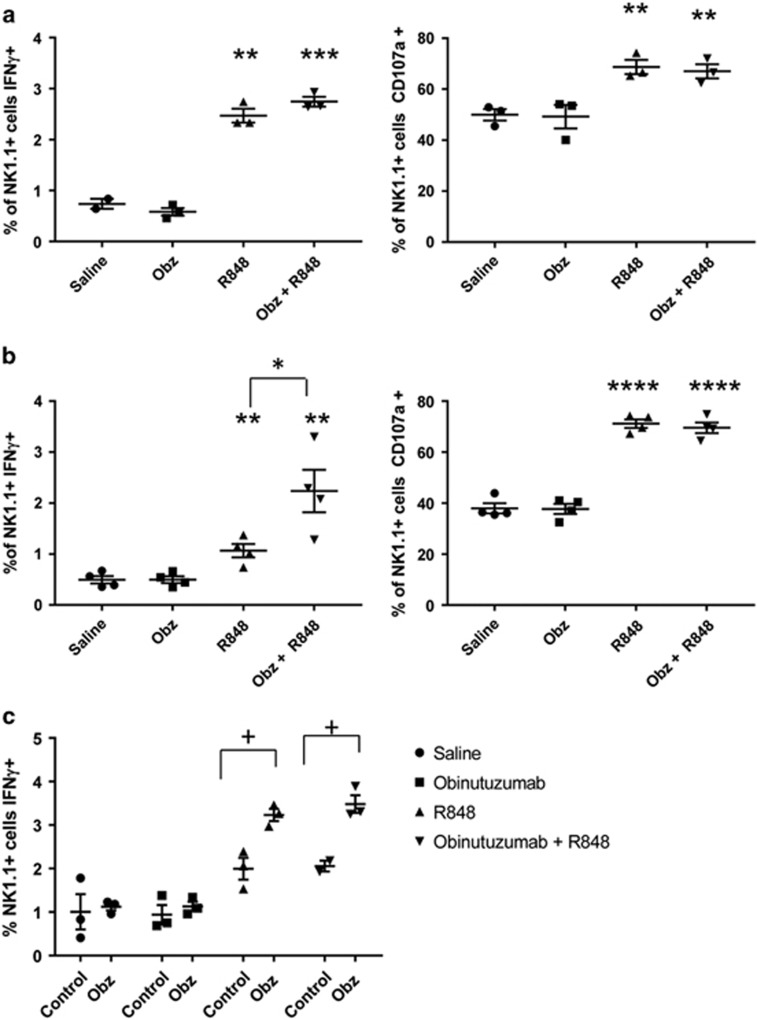
R848 primes murine NK cells *in vivo* for Fc-mediated activity. (**a**–**c**) C57Bl/6 mice were injected with 5 × 10^5^ EL4hCD20 cells i.v. via the tail vein on day 0. Mice received i.p. injections on day 1 of saline (•) or 50 μg obinutuzumab m2a (▪,▾) and i.v. injections of 3 mg/kg R848 via the tail vein on day 1 (▴,▾) (*n*=3 per group). Mice were culled 4 h later (**a** and **c**) or 24 h later (**b**). (**a** and **b**) splenocytes were isolated and cultured for 4 hours in the presence of Golgi transport inhibitors and anti-CD107a PE antibody (right hand panels) at 37 °C before staining for surface NK1.1, intracellular IFNγ (left hand panels) and analyzing by flow cytometry. (**c**) Splenocytes were isolated and cultured on non-tissue cultured treated plates previously coated with 10 μg/ml obinutuzumab m2a or control wells in the presence of golgi transport inhibitors before staining for surface NK1.1, intracellular IFNγ and analyzing by flow cytometry. **P<0.01, ***P<0.001, ****P<0.0001 unpaired student’s *t*- test versus saline control mice unless indicated otherwise,^+^*P*<0.05 unpaired student’s *t*-test versus control wells. Data are representative of two independent experiments.

Having demonstrated the importance of NK cells we examined whether addition of R848 enhanced antitumor activity mediated by NK cells targeted through engagement of obinutuzumab. C57Bl/6 mice bearing EL4hCD20 were culled 4 or 24 h after a single dose of obinutuzumab and/or R848 (Figure 4a and b). Four hours after obinutuzumab monotherapy no activation of NK cells was seen as evidenced by IFNγ production, increased CD107a degranulation (Figure 4a) or upregulation of CD69 and CD137 (Supplementary Figures 8a-b). Given that basal levels of NK cell activation in saline treated tumor bearing mice was high with ~40–50% of NK cells degranulating and expressing CD69 this may be masking detection of obinutuzumab mediated activation. However, a significant upregulation of these markers was seen following R848 administration. Furthermore, by 24 h following therapy there was an increase in IFNγ production (Figure 4b, *P*=0.036, Supplementary Figure 8c) by NK cells from mice that had received obinutuzumab plus R848 compared with mice that had received R848 alone. In order to examine Fc-mediated targeting of NK cells, we cultured splenocytes on obinutuzumab m2a coated plates and analyzed IFNγ production in NK cells. Whereas NK cells from saline or obinutuzumab-treated mice did not respond to engagement of Fc receptors by obinutuzumab, mice that had received R848 significantly upregulated IFNγ production upon binding to obinutuzumab (Figure 4c, *P*<0.05). Furthermore, this upregulation could be reduced by pre-blocking mouse NK cell Fc receptors with an anti-CD16/CD32 antibody (data not shown), suggesting that not all mouse NK cell Fc receptors are blocked *in vivo* by obinutuzumab treatment. Finally in order to show that mouse NK cells are able to mediate killing of EL4hCD20 cells we activated NK cells by culture in high dose IL-2 prior to culture with obinutuzumab opsonized EL4hCD20 cells. Increasing ADCC with increasing effector to target ratios is shown (Supplementary Figure 8d).

### The addition of R848 to obinutuzumab therapy primes tumor-specific T cells that provide protection from tumor rechallenge

**Figure 5:**
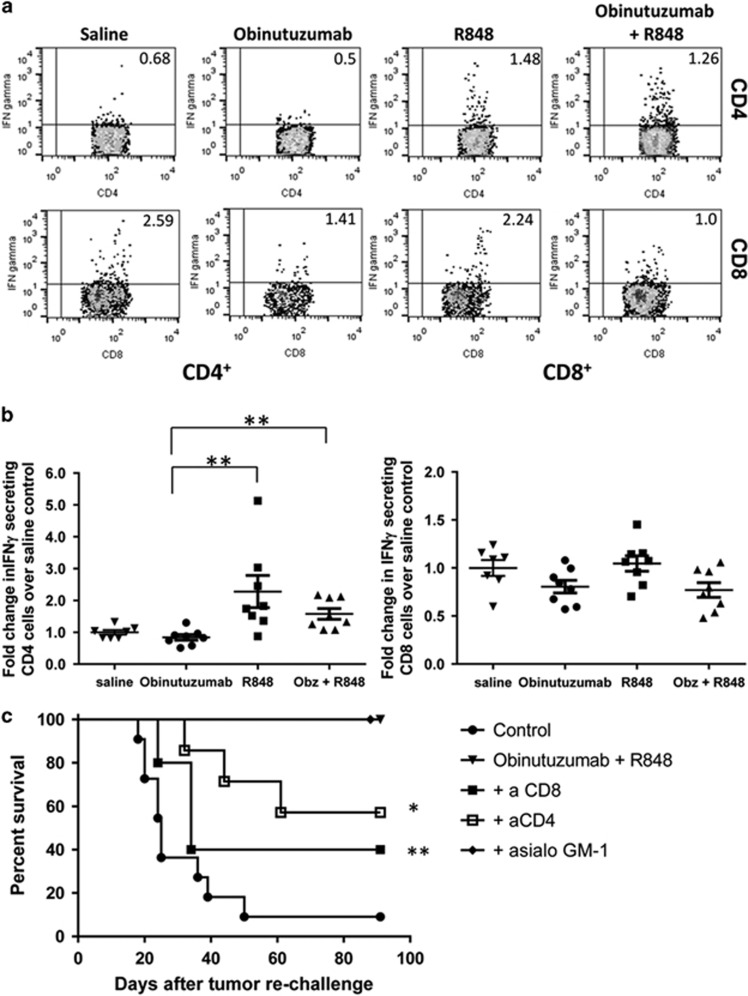
T-cell priming during therapy is important for protection from tumor rechallenge. C57Bl/6 mice were injected with 5 × 10^5^ EL4hCD20 cells i.v. via the tail vein on day 0. Mice received i.p. injections on day 1, 4, 7, 11 and 14 of saline or obinutuzumab m2a and i.v. injections of 3 mg/kg R848 via the tail vein on day 1, 7, 14. Mice were culled on day 18 and splenocytes isolated. Splenocytes were re-stimulated *in vitro* with irradiated EL4hCD20 cells for 5 days prior to a final re-stimulation with irradiated EL4hCD20 cells for 17 h in the presence of brefeldin-A. (**a**) Representative facs plots of CD4^+^ and CD8^+^ T cells producing IFNγ is shown. (The gating strategy is shown in Supplementary Figure 7b). The fold changes in CD4^+^ (left panel) or CD8^+^ (right panel) T cells producing IFNγ in mice receiving obinutuzumab (obz) alone, R848 alone or obinutuzumab plus R848 compared with saline treated mice are shown (*n*=8, data pooled from two independent experiments). **P<0.01, Mann–Whitney test compared with obinutuzumab treated mice. (**c**) C57Bl/6 mice that rejected systemically administered EL4hCD20 cells following treatment starting on day 1 after tumor inoculation, as detailed in Figure 3, were rechallenged with 10^5^ EL4hCD20 cells i.v on day 125 or day 104. Original treatments are shown (• naive mice, ▾ obinutuzumab plus R848, ▪ obinutuzumab plus R848 plus anti-CD8, □ obinutuzumab plus R848 plus anti-CD4, ♦ obinutuzumab plus R848 plus anti-asialo GM1). Kaplan–Meier survival curve is shown (control *n*=11, obinutuzumab+R848 *n*=11, obinutuzumab+R848+anti-CD4 *n*=7, obinutuzumab+R848+anti-CD8 *n*=5, obinutuzumab+R848+anti-asialo GM1 *n*=6). Day 0 represents the day of tumor rechallenge. Data are pooled from one to two independent experiments. *P<0.05, compared with obinutuzumab plus R848 ++*P*<0.01 versus saline (log-rank; Mantel–Cox test).

Given that CD4^+^ but not CD8^+^ T cells appear to have a role in the efficacy of obinutuzumab and R848 combination therapy we investigated whether tumor-specific T cells can be detected early during the course of therapy. An increase in IFNγ producing CD4^+^ T cells was evident at day 18 of combination therapy following *in vitro* restimulation, when compared with obinutuzumab mono-therapy (Figures 5a and b, *P*=0.0011, Mann–Whitney test). R848 mono-therapy also primed the generation of tumor-specific IFNγ-producing CD4^+^ T cells (*P*=0.001) but not CD8^+^ T cells and there was no significant difference between R848 mono-therapy and obinutuzumab plus R848 combination therapy (*P*=0.27). Moreover, no increase in CD4^+^ IFNγ-producing T cells was seen in obinutuzumab mono-therapy treated mice compared with saline treated mice (*P*=0.2). There was a small non-significant decrease in CD8^+^ IFNγ producing T cells with both obinutuzumab mono-therapy (*P*=0.05) and obinutuzumab plus R848 combination therapy (*P*=0.07) compared with controls.

Given that CD4^+^ but not CD8^+^ T cells appear necessary for combination therapy and that only tumor-specific CD4^+^ T cells could be detected, we decided to investigate whether T-cell priming during therapy was critical for protection from tumor rechallenge. LTS from obinutuzumab plus R848 primary therapy that had been depleted of CD4^+^, CD8^+^ T cells or NK cells during the primary therapy were rechallenged ~100 days with EL4hCD20. Whereas mice that had rejected the original tumor in the absence of NK cells rejected the tumor rechallenge, mice which had been depleted of CD4 or CD8 T cells during the primary therapy were significantly more likely to succumb to tumor upon rechallenge (Figure 5c, *P*=0.018, *P*=0.004, respectively). This suggests that whereas CD4^+^ T cells are necessary for therapy, the presence of both CD4^+^ and CD8^+^ T cells during the course of therapy are necessary for the generation of durable antitumor immunity.

### Systemic administration of the TLR7 agonist R848 enhances the therapeutic efficacy of obinutuzumab against established lymphoma in human CD20 transgenic mice

**Figure 6:**
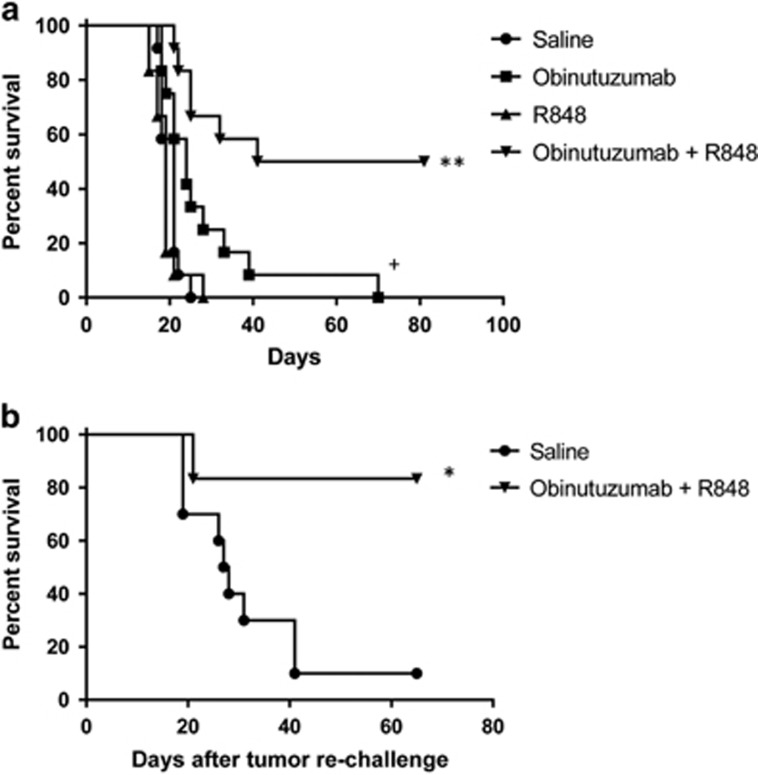
R848 enhances the therapeutic efficacy of obinutuzumab in a longer-term established tumor model in hCD20 transgenic mice. (**a**) hCD20Tg mice were injected with 5 × 10^5^ EL4hCD20 cells i.v. via the tail vein on day 0. Mice received i.p. injections on day 7, 11, 14, 18 and 21 of saline (•) or 50 μg obinutuzumab m2a (▪,▾) and i.v. injections of 3 mg/kg R848 via the tail vein on day 7, 14, 21 and 28 (▴,▾). Kaplan–Meier survival curve is shown of data pooled from two independent experiments (*n*=12 per group). **P<0.01, compared with obinutuzumab (log-rank; Mantel–Cox test), ^+^*P*<0.05, compared with saline (log-rank; Mantel–Cox test). (**b**) Long-term survivors (*n*=6, ▾) or naive hCD20Tg control mice (*n*=10, •) were rechallenged with 10^5^ EL4hCD20 cells i.v on day 83 or 118. Kaplan–Meier survival curve is shown of data pooled from 2 independent experiments. Day 0 represents the day of tumor rechallenge. *P<0.05, compared with control mice (log-rank; Mantel–Cox test).

Having shown that combining obinutuzumab with R848 significantly enhances the survival of hCD20^+^ lymphoma bearing syngeneic mice we established a second model in human CD20 transgenic (hCD20Tg) mice, which express the hCD20 antigen on normal B cells. In this model obinutuzumab targets hCD20^+^ tumor cells and normal B cells, ensuring that hCD20 on tumor cells is not seen as a foreign antigen that would render the tumor more immunogenic. This model is thus more akin to the clinical situation. In order to establish if combination therapy was effective against higher tumor burden, treatment was delayed to 7 days post-systemic inoculation of EL4hCD20 cells. Although obinutuzumab significantly increased survival compared with saline controls (*P*=0.02) there were no LTS (Figure 6a). However, combining R848 with obinutuzumab significantly increased survival compared with obinutuzumab mono-therapy (*P*=0.003) with 6 of 12 mice LTS (Figure 6a). No increase in survival was seen with R848 mono-therapy compared with controls. Furthermore, five of six obinutuzumab plus R848 combination therapy LTS rejected tumor rechallenge (Figure 6b, *P*=0.01), suggesting induction of immunological memory.

### R848 enhances NK cell-mediated ADCC of primary B-CLL cells

**Figure 7:**
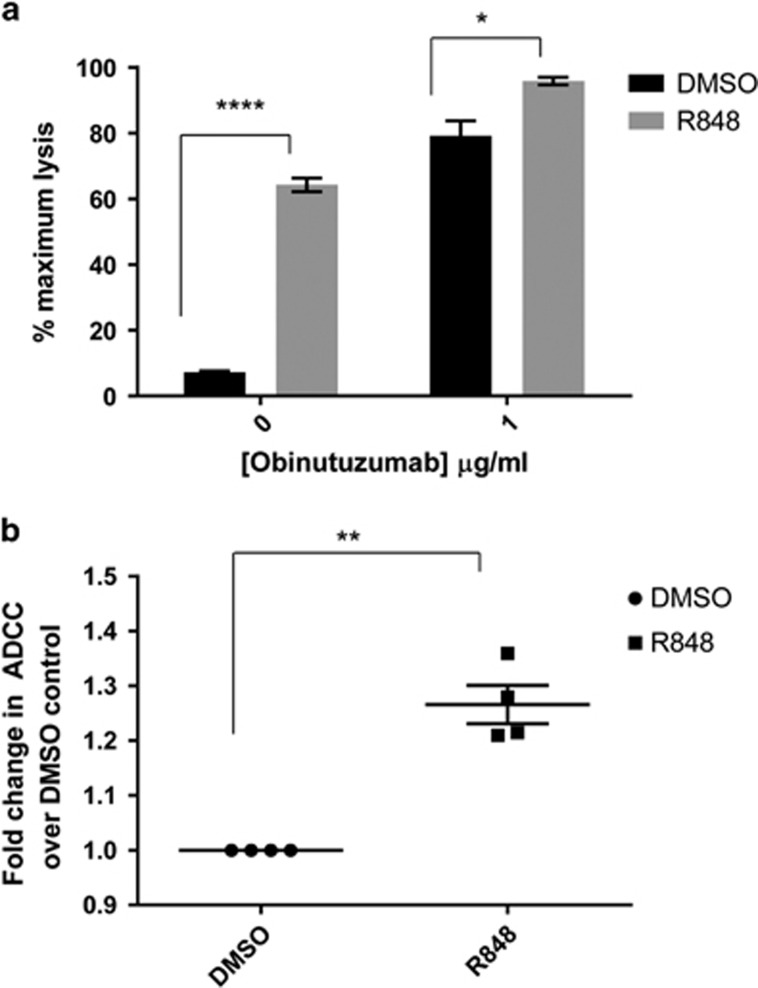
R848 enhances NK cell ADCC against primary B-CLL tumor cells. Human PBMC from healthy donors were activated for 20 h with 30 μM R848 (▪) or DMSO (•) equivalent. NK cells were isolated and cultured with 20,000 calcein-AM labeled B-CLL cells for 2 h in the presence or absence of 1 μg/ml obinutuzumab at a 10:1 effector: target ratio. % ADCC was measured by measurement of calcein release into the supernatant compared with maximum lysis in 4% Triton. (**a**) ADCC plotted as % maximum lysis as detailed in materials and methods for one representative CLL sample of three performed in triplicate wells. Data are plotted as mean plus s.e.m. (**b**) Fold change in ADCC with R848 treated NK cells compared with DMSO treated NK cells against 1 μg/ml obinutuzumab opsonized B-CLL cells. Data are plotted for two healthy donor NK cells against three different B-CLL samples. *P*<0.01, *P*<0.001, ****P<0.0001, unpaired students *t*-test assuming unequal variance.

Given that the R848-mediated enhancement of the antitumor efficacy of obinutuzumab was partially dependent on NK cells we investigated whether R848 could enhance ADCC against primary B-CLL cells. A statistically significant increase in NK cell-mediated ADCC against obinutuzumab-opsonized B-CLL cells was seen (Figure 7a, *P*=0.02), although R848 also significantly increased non-specific antibody-independent killing of B-CLL cells. On average R848 pre-treatment increased ADCC against obinutuzumab opsonized B-CLL cells by 1.3-fold (Figure 7b).

## Discussion

We demonstrate that addition of systemically administered TLR7 agonist R848 to obinutuzumab therapy can significantly enhance long-term survival in syngeneic models of lymphoma. Importantly, we also demonstrated improved survival in lymphoma bearing hCD20Tg mice that more closely mimic the patient population with expression of hCD20 on B cells.

This is the first report that the antitumor efficacy of obinutuzumab can be significantly improved by systemic administration of a TLR7 agonist. Although R848 can enhance non-specific NK cell cytotoxicity, ADCC and ADCP the *in vivo* mechanism of action appeared to be solely dependent on NK cells and CD4^+^ T cells. A type I anti-CD20 mAb was previously shown to eradicate EL4hCD20 cells in C57Bl/6 mice by the same mechanism.^19, 20^ Taken together with data that R848 and other TLR agonists can increase levels of activating FcγR but decrease levels of the inhibitory FcγRIIb,^21, 22^ this suggests that R848 is potentiating obinutuzumab effector mechanisms, in particular ADCC by NK cells, rather than priming other immunological pathways. Indeed, *in vivo* priming of NK cells with R848 was necessary for enhanced IFNγ production by NK cells upon engagement of Fc receptors by obinutuzumab. Furthermore, R848 was able to enhance ADCC against primary B-CLL cells and lymphoma cell lines as shown for other TLR agonists for a variety of tumor-targeting mAbs.^21, 23, 24, 25^ The TLR3 agonist poly (I:C) has been shown to extend survival in a lymphoma model in combination with anti-mouse CD20 mAb, but the mechanistic role of T cells and NK cells were not studied.^26^ Whereas the antitumor efficacy of rituximab has previously been enhanced by intra-tumoral administration of the TLR9 agonist CpG, systemic administration of CpG was not effective and as a result only local tumor control secondary to local administration was seen.^27, 28^ In this model, NK cells were again shown to be the major effector cell with no apparent role for T cells or macrophages: no loss of therapy was seen in clodronate-liposome-treated mice. However, when a CpG oligonucleotide 1018 ISS was combined with rituximab subcutaneously in a phase II clinical trial the response rate was similar to that reported for rituximab alone.^29^ Although infiltration of CD8^+^ T cells and macrophages into tumor was observed, these data suggest that systemic administration of TLR agonists may be beneficial for sustained antitumor responses.

The relative role of the different anti-CD20 mAb effector mechanisms in eradicating malignant disease in patients remains unclear, although there is evidence that Fc-mediated mechanisms are important as patients with FcγR polymorphisms, which bind the rituximab Fc region with higher affinity respond better to rituximab therapy.^30^ In murine models some studies report a primary role for phagocytic mechanisms.^26, 31, 32, 33^ However, others have demonstrated the importance of ADCC by NK cells and these studies used a similar EL4hCD20 model to ours,^19, 20^ suggesting that the mechanism of action may vary depending on the tumor model, location and antibody isotype. Here we have demonstrated that for obinutuzumab, when given in combination with a systemic TLR7 agonist, long-term tumor-free survival appears to be dependent on NK cells and CD4^+^ T cells with macrophages and monocytes only able to mediate short-term increases in survival. Whereas TLRs have been shown to activate macrophages, ligation of high-affinity FcγRs by IgG immune complexes can downregulate TLR signaling in macrophages. Thus, we cannot exclude the possibility that obinutuzumab-binding FcγR on macrophages may be abrogating the benefits of TLR7 agonism.^34^ TLR agonists can potentially have a diverse role in activating the immune system and thus may enhance antibody-mediated tumor cell killing by numerous different mechanisms. Given that both anti-CD20 mAb^20^ and R848 therapy^35, 36^ can prime Th1 immune responses through NK cell-mediated IFNγ release and production of IL-12 by dendritic cell, and that TLR7 agonism^37, 38^ leads to direct induction of CD4 T-cell proliferation and stimulation of CD4 T cells through activation of antigen-presenting cell, it is likely that obinutuzumab and R848 act in synergy to activate tumor-specific CD4^+^ T cells. It was previously shown in wild-type mice bearing EL4hCD20 that protection from tumor rechallenge post anti-CD20 mAb therapy was restricted to hCD20^+^ tumors, suggesting a vaccination effect.^33^ Here we show that hCD20Tg mice, which express hCD20 on B cells and are tolerized to hCD20, are also protected from tumor rechallenge post-combination therapy. This suggests that the addition of an immunostimulatory TLR7 agonist is able to extend the repertoire of tumor-specific T cells to non-foreign antigens.

Given that combination therapy led to protection from tumor rechallenge, it was surprising that there was no role for CD8^+^ T cells in primary therapy. Although obinutuzumab can induce immunogenic cell death,^12^ in this model enhanced NK cell-mediated ADCC may eradicate tumor before establishment of a tumor-specific CD8-mediated T-cell response. However, although IFNγ secreting tumor-specific CD8^+^ T cells were not detected post therapy, depletion of CD8^+^ T cells during the course of therapy appeared critical for generation of immunological memory as over half of these mice were not protected from rechallenge, in agreement with a previous report.^19^ Given that CD4^+^ T cell depletion also reduced protection from rechallenge it is likely that both T-cell subsets are acting in concert to prime long-term immunological memory.^39^

The novel glycoengineered type II anti-CD20 mAb obinutuzumab has previously been shown to be highly effective *in vitro*^4, 5, 6, 40^ and in xenograft models.^41, 42, 43^ Here, we demonstrate the ability of obinutuzumab to treat lymphoma in mice with an intact immune system. Although the majority of mice had increased survival with obinutuzumab mono-therapy, long-term survival was low. Given that the glycoengineering of obinutuzumab enhances its affinity to FcγRIIIa and that mouse FcγRIV, the murine homolog of human FcγRIIIa, is not expressed on mouse NK cells,^44^ it is impossible to determine the effects of enhanced ADCC by glycoengineered obinutuzumab in this model. However, our data demonstrate that combination therapy with a TLR7 agonist can significantly enhance long-term survival. Moreover, a similar enhanced efficacy was seen when the non glyco-engineered type I antibody rituximab was combined with R848, suggesting that TLR7 agonists can potentiate the efficacy of all anti-CD20 antibodies and not just glycoengineered type II anti-CD20 antibodies.

The impact of immune-modulatory therapies on clinical practice is currently being realized with durable remissions in a number of solid cancers post immune checkpoint blockade.^13^ Although to date the role of immune checkpoint molecules in lymphoma is still emerging, the data presented here are encouraging and suggest that the antitumor activity of anti-CD20 antibodies can be enhanced by combination approaches with immune-stimulatory agents. Strategies to improve the efficacy of anti-CD20 mAb therapy and provide enhanced antitumor immunity would appear highly likely to lead to improved patient survival. There is currently an unmet need to develop combination approaches to enhance anti-CD20 antibodies through immune modulation and systemic administration of TLR agonists, in particular those targeting TLR7, may be highly effective in combination with the novel anti-CD20 mAb obinutuzumab and appear worthy of further clinical investigation.
