# Supplementary material for: A TLR7 agonist enhances the antitumor efficacy of obinutuzumab in murine lymphoma models via NK cells and CD4 T cells
Source: Leukemia. 2017 Jan 3;31(7):1611–21. doi: 10.1038/leu.2016.352 (PMC5508079; doi:10.1038/leu.2016.352)
Supplement: Supplementary Information [file leu2016352x1.docx]

**Supplementary Materials and Methods**

*Generation of EL4 expressing human CD20*

EL4hCD20 cells were generated as described below. Human CD20 was subcloned from the pBABEhCD20 vector (gift from Prof M Cragg and Dr C Chan, University of Southampton, UK) as a *Bam HI*, *Not I* fragment by PCR and subsequent TOPO TA cloning into the pCR4 vector (Invitrogen). hCD20 was subcloned into the retroviral vector rKat as a *Bam HI*, *Not I* fragment. Amphotrophic retroviral supernatant was generated by transient transfection of 293T[^16^](#_ENREF_16) and EL4 were infected by centrifugation of cells in viral supernatant at 1200xg for 1.5 hours on retroviral supernatant loaded retronectin coated plates (6-well plates, 10μg/mL retronectin, Takara-Mirus-Bio, USA). EL4-hCD20 was generated by three rounds of single-cell cloning by limiting dilution and tested for human CD20 expression by staining with 1ug Rituximab (Roche, Christie Hospital Pharmacy) followed by PE-anti human IgG (gamma chain specific) (Sigma-Aldrich) for 30 minutes on ice or anti-human CD20 APC (Supplementary Figure 1). Samples were acquired on a FACS Calibur and analyzed using FlowJo software (Treestar, Miltenyi Biotech, UK).

*In vivo studies*

Mice were housed under specific pathogen free conditions in Tecniplast 1284 IVC cages holding a maximum of 7 animals on aspenchips-2 bedding with sizzlenest nesting material and a cardboard tunnel on a 12/12 light/dark cycle. Mice were given filtered water and fed ad-lib on Teklad Global 19% protein extruded rodent diet. Group sizes were based on previous power calculations following discussion with a statistician. Female C57Bl/6 mice were used at around 8 weeks of age at around 17-20g in weight. Both male and female hCD20Tg mice were used at around 12-20 weeks of age. hCD20Tg mice were allocated to groups to ensure an equal number of male and female mice and age matched mice. Assessment of weights and endpoints related to tumor burden was performed by technical staff to ensure a lack of bias. Mice were excluded from survival analyses only in rare cases if culled due to non-tumour related endpoints such as wounds due to fighting.

*Flow cytometry to confirm cellular depletion in vivo*

Heparanized tail bleeds were taken from the tail vein four days after start of treatment to deplete T-cells and NK cells to confirm cellular depletion. Briefly, red blood cells were lysed in Pharmlyse solution (Becton-Dickinson, UK) and cells incubated with rat anti-CD16/CD32 Fc block (#16-0161) for 15 minutes on ice. Cells were incubated with Gr-1 FITC (#11-5931), CD4-PE (#12-0042), CD8-PE-Cy7 (#25-0081), CD11b-APC (#17-0112) or NK1.1-FITC (#11-5941), Ly-6B.2-PE (Miltenyi Biotech #130-102-857), F4/80-PerCP-Cy5.5 (#45-4801), CD49b APC (Miltenyi Biotech, #130-102-337) for 30 minutes on ice, washed in 1% FCS/PBS and samples acquired on a FACS Calibur and analyzed using FlowJo software. The number of cells per ml blood was enumerated by the addition of CountBright™ beads (Invitrogen) during the acquisition process and the assumption that 1g of blood is equivalent to 1ml blood.

*Human ADCC assays*

PBMC were isolated from heparinized peripheral blood by density centrifugation on lymphoprep (Axis-Shield, UK) and pre-treated for 20 hours in 30μM R848/ DMSO control + 20IU/ml IL-2. Isolated NK cells (NK cell isolation kit, Miltenyi-Biotech) were cultured for 2 hours with obinutuzumab opsonized calcein-AM labeled (Molecular Probes) Raji or primary B-CLL cells. Cytotoxicity was determined by measuring calcein release using a fluorescent plate reader and percentage specific lysis was determined compared to lysis in 4% triton-X100 using the formula:

(observed release – spontaneous release) _x 100_

(maximum release – spontaneous release)

*Mouse NK cell ADCC assays*

NK cells were isolated from C57Bl/6 mice spleens using the NK cell isolation kit (Miltenyi Biotech). NK cells were cultured at 10^6^/ml in 3000IU/ml human recombinant IL-2 for two weeks (Proleukin, Christie Hospital NHS pharmacy, UK). NK cells were cultured with EL4hCD20 cells opsonized with 0.1-1μg/ml obinutuzumab m2a for 4 hours at decreasing effector: target ratios and cytotoxicity determined using the LDH cytotoxicity detection kit^PLUS^ as per the manufacturer’s instructions (Roche, UK). Percentage maximum lysis was determined compared to lysis in 4% triton-X100 and percentage specific lysis was calculated by subtraction of percentage maximum lysis against EL4hCD20 alone.

*Intracellular staining for IFNγ*

Splenocytes were incubated with rat anti-CD16/CD32, stained with αCD8α-FITC (#11-0081), αCD4-PE (#12-0042) or αNK1.1PE-Cy7 (#25-5941), αCD69-PE (#12-0691), αCD137APC (#17-1371) as appropriate on ice for 30 min, fixed/permeabilized (FoxP3 staining buffer set, eBioscience) and incubated with αIFNγ-APC on ice for 30 min. Following washing samples were acquired on a FACS Calibur and analyzed using FlowJo software.

*Mouse IFNγ secretion*

NK cells were isolated from treated C57Bl/6 mice spleens using the NK cell isolation kit (Miltenyi Biotech). 10^5^ NK cells were cultured in 200μl media on non-tissue cultured treated plates previously coated with 10μg/ml obinutuzumab m2a or control borate buffer. After 20 hours IFNγ was measured in the supernatant using an IFNγ ELISA set (Diaclone, 2B Scientific, UK) as per the manufacturers instructions.

*ADCP assay*

Neutrophils were isolated from the bone marrow of C57Bl/6 mice using the mouse neutrophil isolation kit (Miltenyi Biotech) and pre-treated with 20μM R848 for 2hrs at 37°C. PKH26 (Sigma-Aldrich) labeled EL4hCD20 cells opsonized with 20μg/ml obinutuzumab were added at 1:1 ratio to give a final concentration of 10μM R848 and 10μg/ml obinutuzumab. Cells were incubated for 20hrs at 37°C, stained with CD11b-APC (#17-0112), washed and acquired on a FACS Calibur and analyzed using FlowJo software for the percentage uptake of PKH26^+^ tumor cells by CD11b^+^ neutrophils.

*Statistical Analysis*

All statistical analysis was undertaken using GraphPad PRISM software. For in vitro experiments data was assumed to be normally distributed. The 2-way ANOVA was used to compare outcome data from more than 1 time point following more than 1 intervention. Unpaired t-testing was also used to compare outcomes from 1 time point following a single intervention. All error bars represent standard error of the mean. All P-values are reported as; ns p>0.05, * p≤0.05, **p≤0.01 ***p≤0.001, **** p≤0.0001. Equal variances were assumed unless data was pooled from more than one donor/patient sample. Survival analyses were performed using log-rank, mantel-cox tests. Where data was assumed to be non-normally distributed a non-parametric Mann-Whitney U test was performed.

**Supplementary Figure Legends**

**Supplementary Figure 1. Expression of human CD20 by EL4hCD20 cells**

EL4hCD20 cells were generated as described in Supplementary methods. EL4 (red histograms) and EL4hCD20 cells (blue histograms) were stained with anti-human CD20 APC (a) or rituximab followed by anti-human IgG (gamma chain specific)-PE (b). Representative facs plots are shown.

**Supplementary Figure 2. Systemic administration of the TLR7 agonist R848 activates NK cells and T-cells in vivo.**

(a and b) C57Bl/6 mice (n=5) received an iv injection of R848 (3mg/kg) or DMSO control via the tail vein. 20hr later mice were culled and splenocytes were analysed for expression of NK1.1, CD49b, CD3, CD4, CD8 and CD69 by flow cytometry. (a) Percentage of NK1.1^+^, CD49b^+^, CD3^+^CD49b^+^, CD4^+^, and CD8^+^ cells which are CD69^+^cells is shown. ** P<0.01, Mann-Whitney test. (b) Representative flow cytometry histograms for CD69 staining in NK1.1^+^, CD49b^+^, CD3^+^, CD4^+^ and CD8^+^ cells is shown. (c) Neutrophils were isolated from the bone marrow of C57Bl/6 mice and cultured for 2hr with or without 10µM R848. Neutrophils were then cultured for a further 20 hours with PKH26 labeled EL4hCD20 cells in the presence or absence (no treatment, NT) of obinutuzumab before labeling with anti-CD11b and analysis by flow cytometry. Representative flow cytometry histograms for CD11b staining is shown.

**Supplementary Figure 3. Detection of tumour specific T-cells in LTS post obinutuzumab and R848 combination therapy.**

LTS following obinutuzumab and R848 therapy which had rejected a tumor rechallenge were rechallenged with EL4hCD20 cells on day 212. Seven days later mice were culled and splenocytes were either re-stimulated in vitro with irradiated EL4hCD20 cells for 17 hours in the presence of brefeldin-A following a five days *in vitro* re-stimulation with irradiated EL4hCD20 cells. The % of CD4 T-cells (left-panel) and CD8 T-cells (right panel) producing IFNγ is shown. * P<0.05, Mann-Whitney-U compared to naïve untreated C57Bl/6 mice.

**Supplementary Figure 4. Depletion of immune subsets following antibody administration in vivo.**

(a) C57Bl/6 mice received saline, 500 µg anti-CD8, 250 µg anti-CD4, 100 µg anti-NK1.1 or 50µl anti-asialo-GM1 ip on day 1 of therapy. Four days later tail bleeds were taken, red blood cells lysed and stained with CD4-PE, CD8 PE-Cy7 or NK1.1 FITC and CD49b APC. Representative facs plots are shown. (b) C57Bl/6 mice were injected with 5x10^5^ EL4hCD20 cells i.v. via the tail vein on day 0. Depletion antibodies were given i.p. four hours prior to mAb on day 1, 4, 7, 11 (100 µg αNK1.1, 50µl α asialo-GM1) or day 1 and 8 (250 µg αCD4). Mice received saline i.p. on day 1, 4, 7, 11 and 14 or 50µg obinutuzumab m2a and i.v injections of 3mg/kg R848 via the tail vein on day 1, 7, 14 and 21. The number of NK1.1^+^ F4/80^-^ cells/ml blood at day 4 is shown for mice treated as described in Figure 3a with obinutuzumab + R848 + αCD4 + α asialo-GM1 or mice treated as described in Figure 3d with obinutuzumab + R848 + αCD4 + αNK1.1. **P<0.01 (Mann-Whitney test).

**Supplementary Figure 5. R848 leads to an increase in circulating NK cells three days after initiation of therapy.**

C57Bl/6 mice were injected with 5x10^5^ EL4hCD20 cells i.v. via the tail vein on day 0. Mice received i.p. injections on day 1 of saline (●) or 50µg obinutuzumab m2a (■,▼) and i.v. injections of 3mg/kg R848 via the tail vein on day 1 ( ▲,▼). Tail bleeds were taken on day 4 and weighed and the number of CD4^+^, CD8^+^, NK1.1^+^, CD11b^lo^, F4/80^+^ and CD11b^Hi^Gr-1^Hi^ cells was analysed by flow cytometry. The number of cells/ml of blood was calculated using CountBright™ beads and the relative change in absolute cell numbers was calculated compared saline control mice for each individual mouse. Data is pooled from 2-4 individual experiments. +P=0.06, * P<0.05, **P<0.01, Mann-Whitney-U compared to obinutuzumab m2a treated mice.

**Supplementary Figure 6. Gating strategy for NK1.1^+^F4/80^+^ cells**

(a) Tail bleeds from mice treated as described in Figure 3B were stained with NK1.1 APC and F4/80 PerCP Cy5.5 as described in the methods. In order to determine the percentage of cells expressing NK1.1 and F4/80 a gate was drawn around the lives cells using FSC/SSC. Cells were gated as NK1.1^+^, F4/80^-^, NK1.1^+^, F4/80^+^, NK1.1^-^, F4/80^+^ as shown for a representative mouse. (b) Tail bleeds from mice treated as described in Figure 3c were stained with NK1.1 FITC and F4/80 PerCP Cy5.5 as described in the methods. The gating strategy is shown for a representative mouse.

**Supplementary Figure 7. Gating strategy for IFNγ secreting T-cells.**

Gating strategy for IFNγ secreting T-cells shown in Figure 5A. Splenocytes were isolated from mice treated as described in Figure 4a, re-stimulated in vitro with irradiated EL4hCD20 cells for five days prior to a final re-stimulation with irradiated EL4hCD20 cells for 17 hours in the presence of brefeldin-A. Cells were stained with CD8-FITC, CD4 PerCP Cy5.5 and IFNγ APC, gated on lymphocytes using FSC/SCC and CD4^+^ CD8^-^ or CD4^-^CD8^+^ cells prior to calculating the number of IFNγ secreting CD4/CD8 T-cells.

**Supplementary Figure 8. Activation of NK cells 4 and 24 hours post obinutuzumab and R848 administration.**

C57Bl/6 mice were injected with 5x10^5^ EL4hCD20 cells i.v. via the tail vein on day 0. Mice received i.p. injections on day 1 of saline (●) or 50µg obinutuzumab m2a (■,▼) and i.v. injections of 3mg/kg R848 via the tail vein on day 1 ( ▲,▼) (n=3 per group). Mice were culled 4 hours (a) or 24 hr (b and c) later. Splenocytes were isolated, stained for NK1.1, CD69 and CD137 and analyzed by flow cytometry. (a and b) Percentage of NK1.1^+^ cells expressing CD69 or CD137 is shown. (c) 4 mice were treated as described in b, splenocytes from pairs of two mice were pooled and an NK cell enrichment performed. IFNγ secretion from 10^5^ cells cultured for 20 hours in 200μl media on non-tissue cultured treated plates previously coated with 10μg/ml obinutuzumab m2a or control wells was measured by ELISA. Where cell numbers permitted samples were run in duplicate. * P<0.05, **P<0.01, ***P<0.001, ****P<0.0001 unpaired students t-test versus saline control mice. (d) NK cells were isolated from C57Bl/6 splenocytes and cultured in 3000IU/ml IL-2 for two weeks. NK cells were cultured with 5000 EL4hCD20 cells for 4hr in the presence or absence of varying concentrations of obinutuzumab m2a at decreasing effector: target ratios. % ADCC was measured by LDH release into the supernatant compared to maximum lysis in 4% Triton.
