# Supplementary figures and images for: A TLR7 agonist enhances the antitumor efficacy of obinutuzumab in murine lymphoma models via NK cells and CD4 T cells
Source: Leukemia. 2017 Jan 3;31(7):1611–21. doi: 10.1038/leu.2016.352 (PMC5508079; doi:10.1038/leu.2016.352)

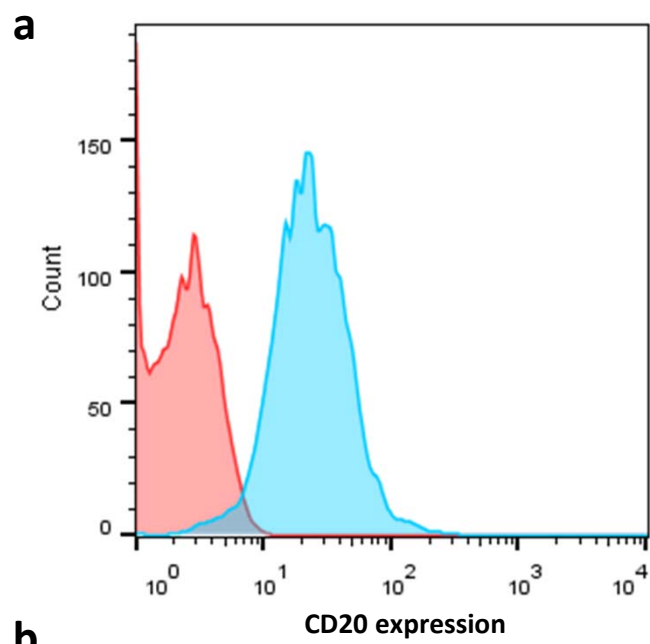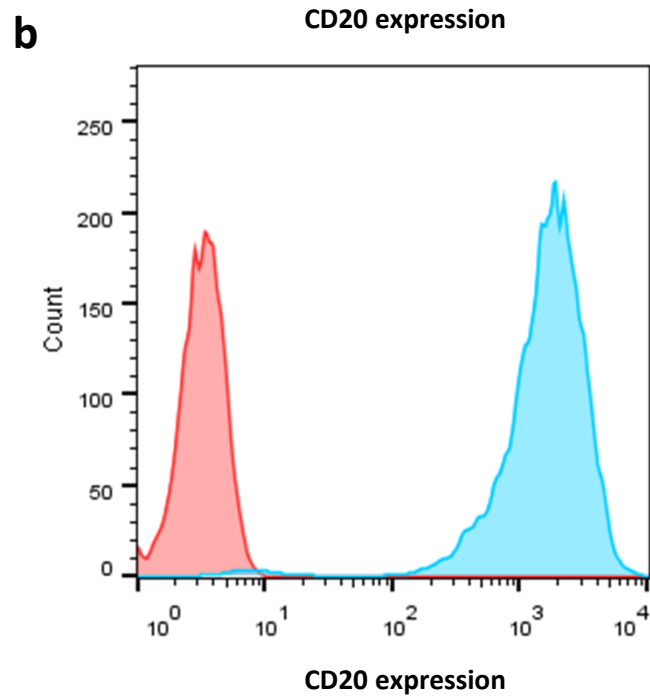

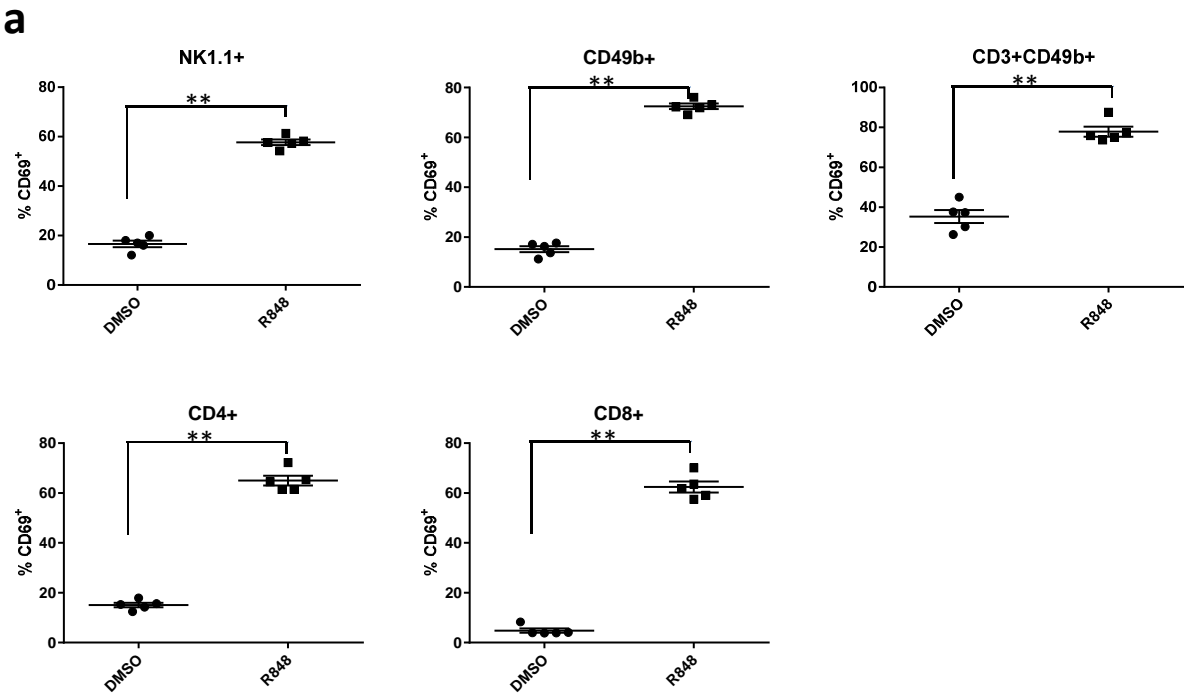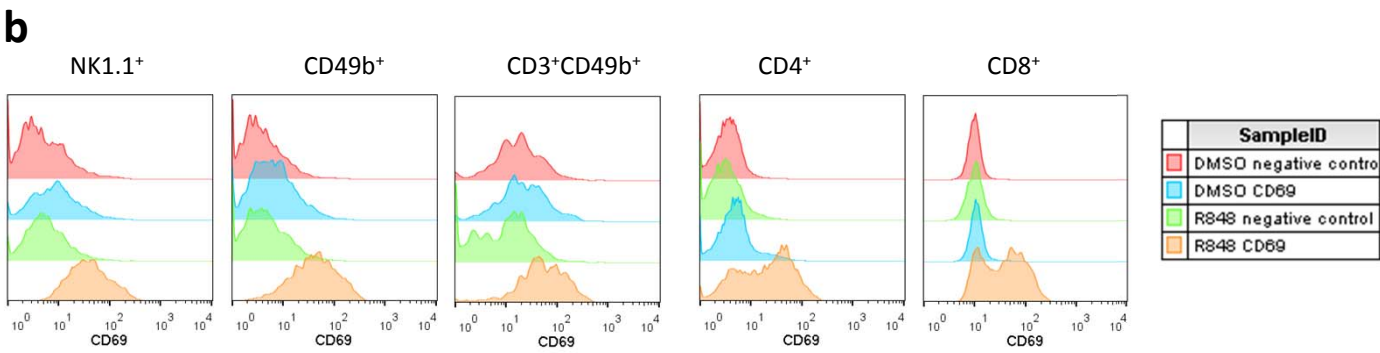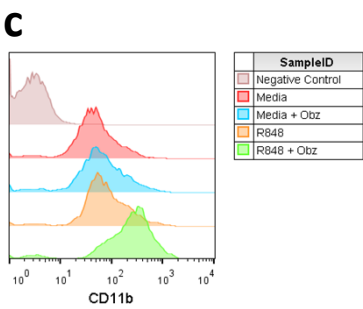

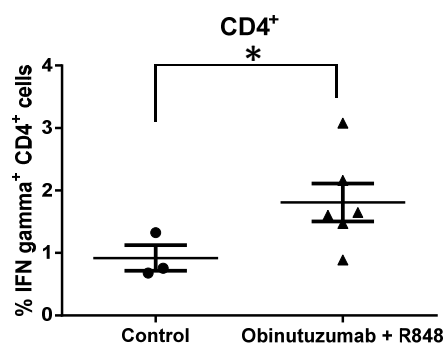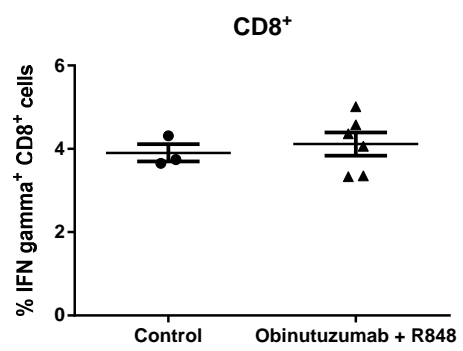

**a**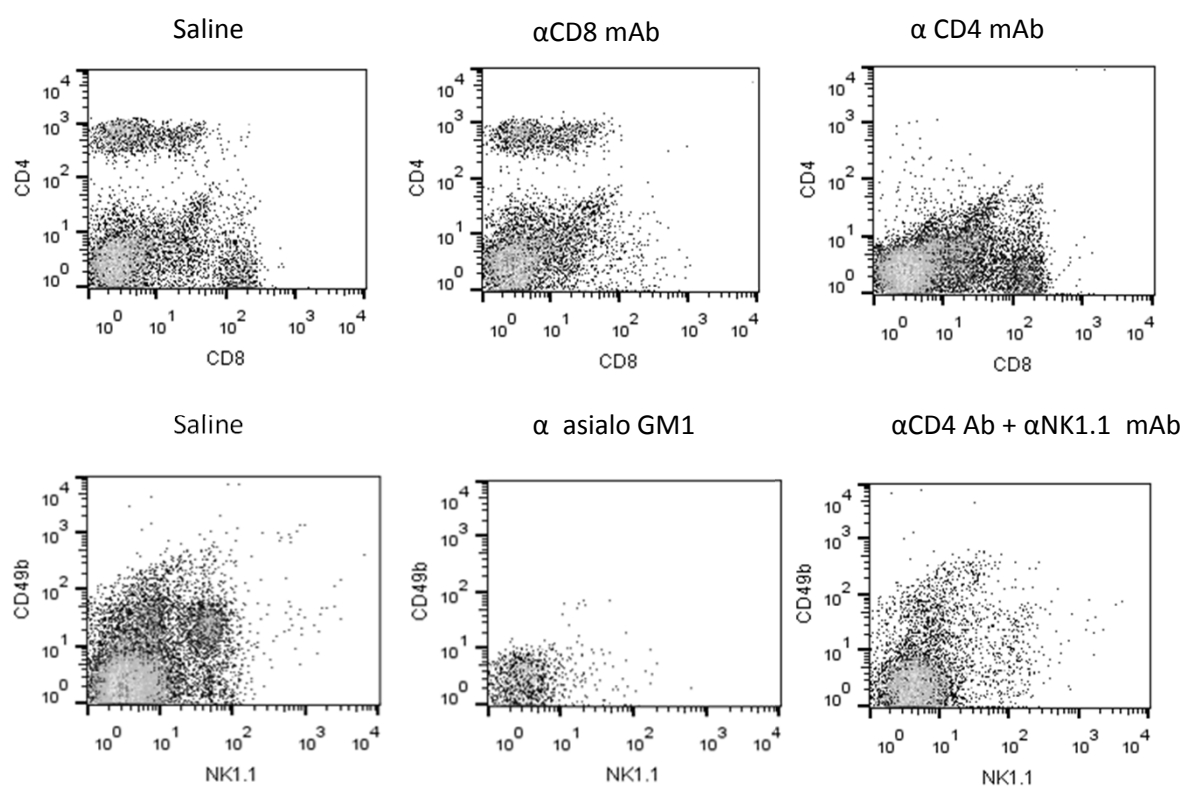**b**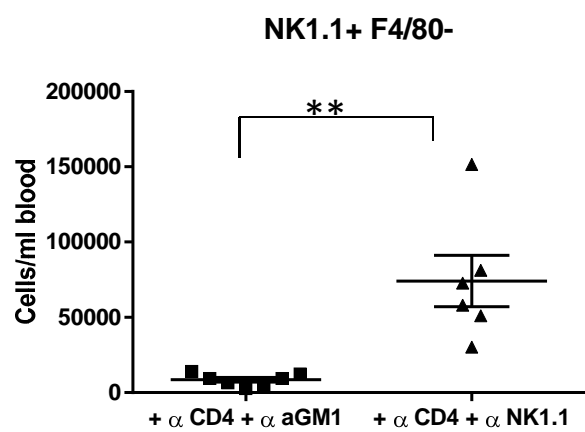

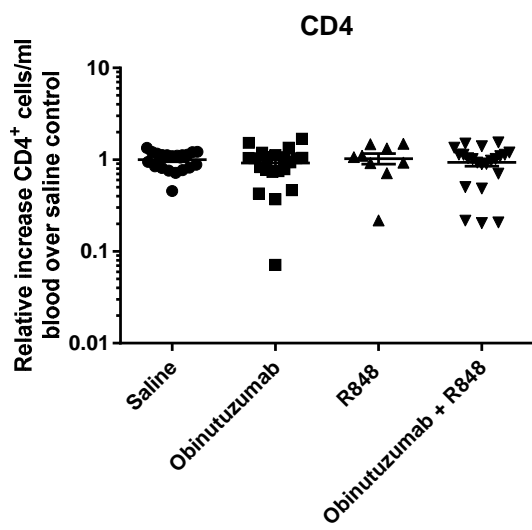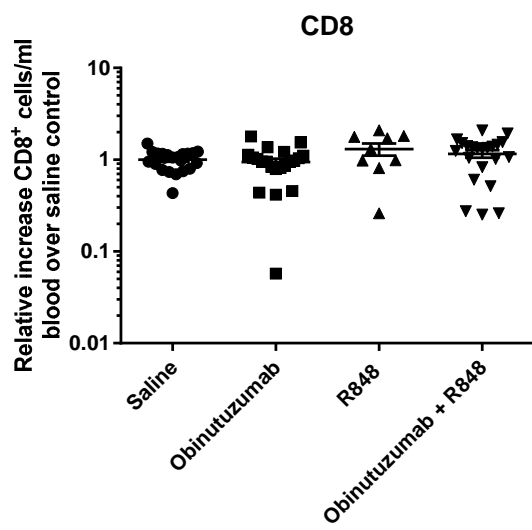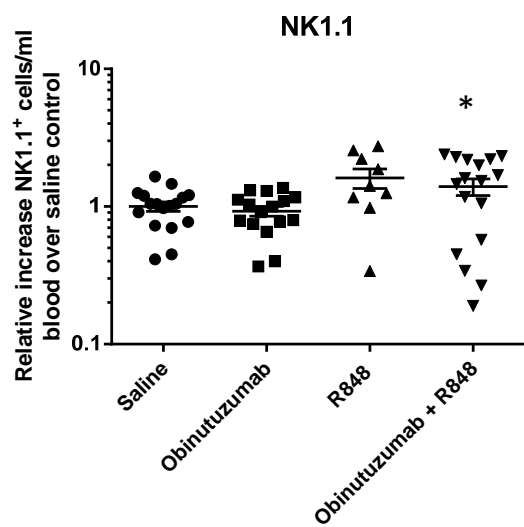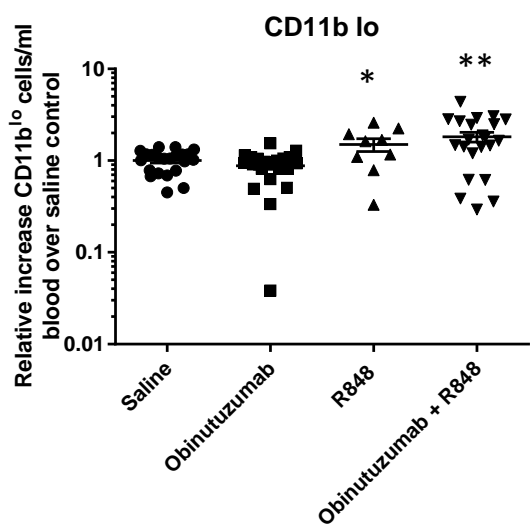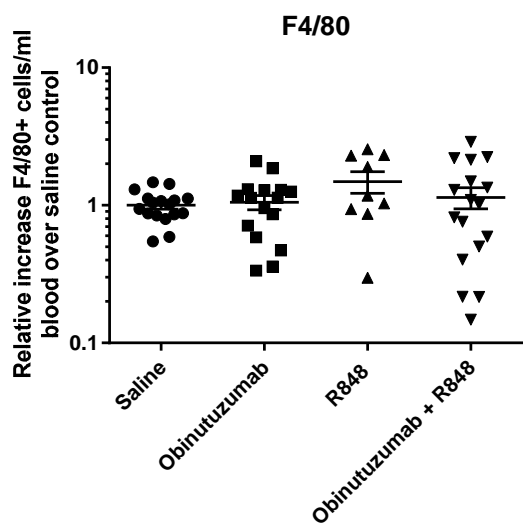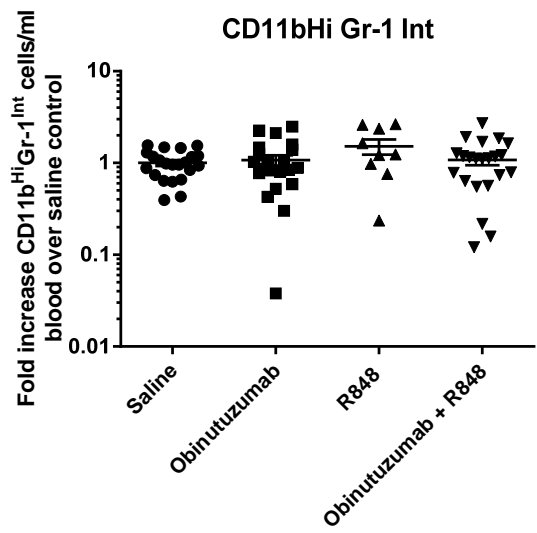

**a**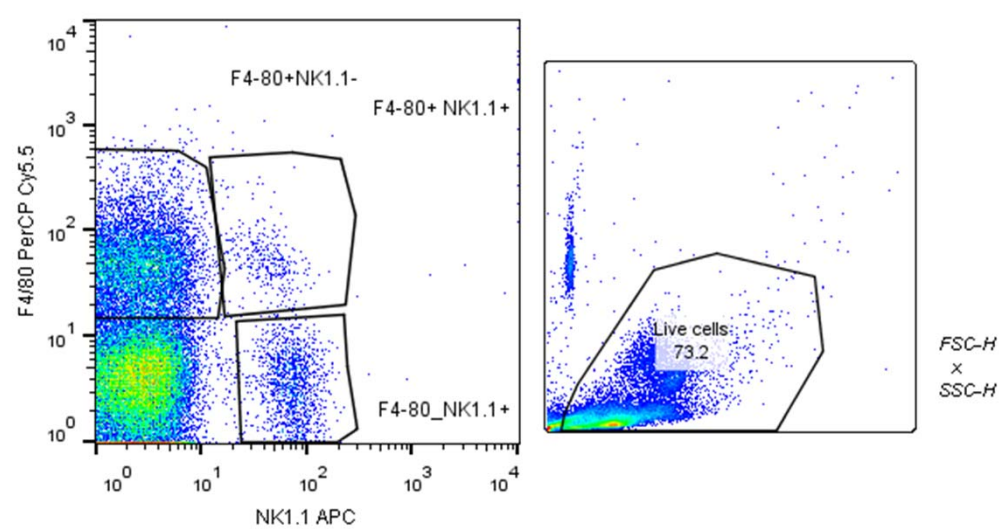**b**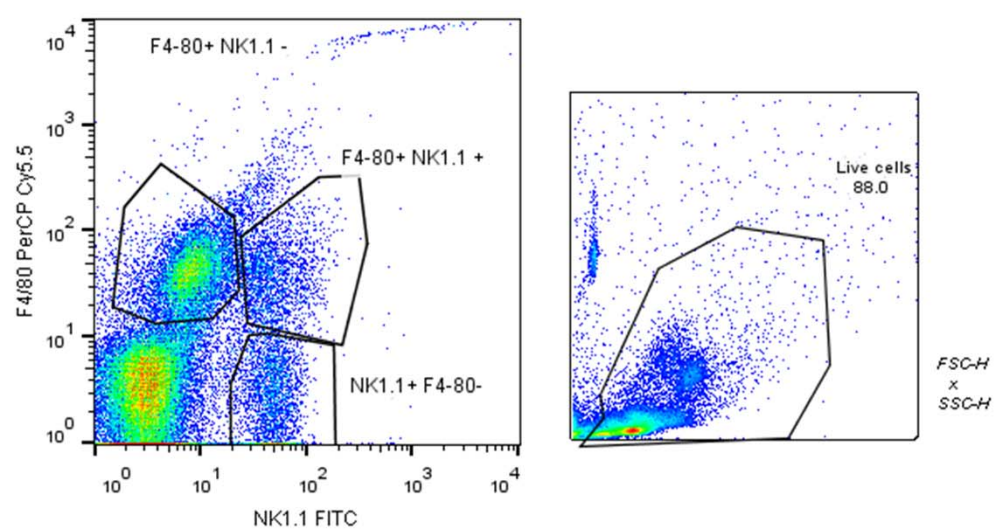

CD4

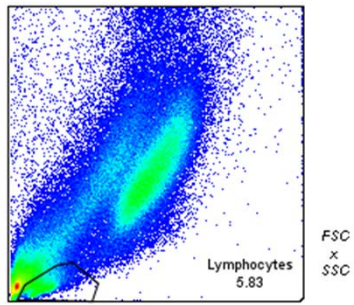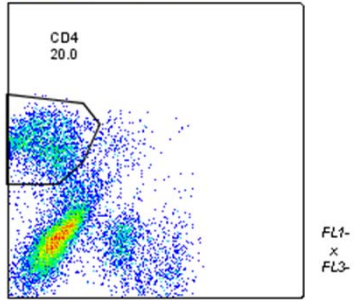

CD8

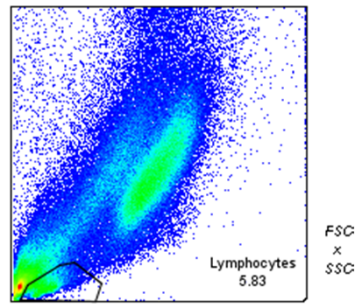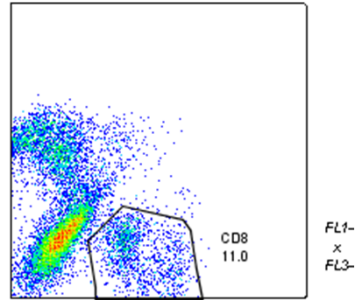

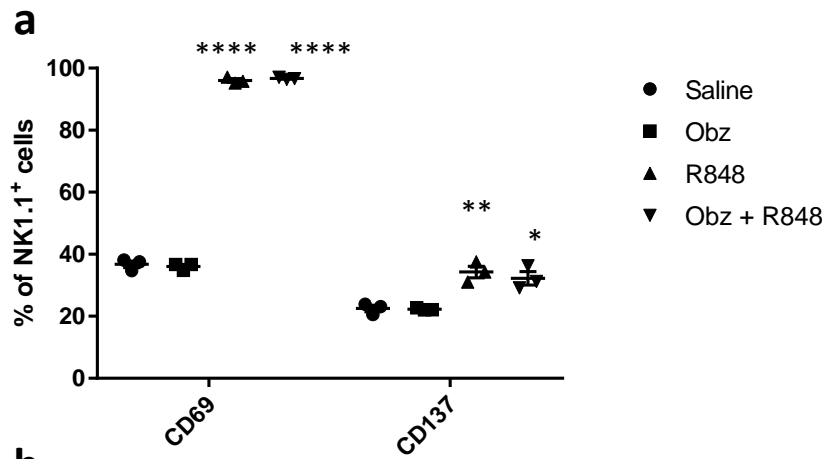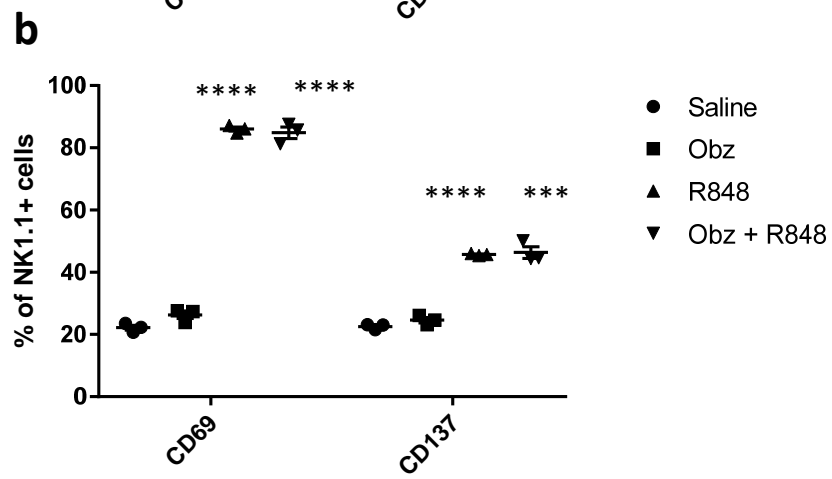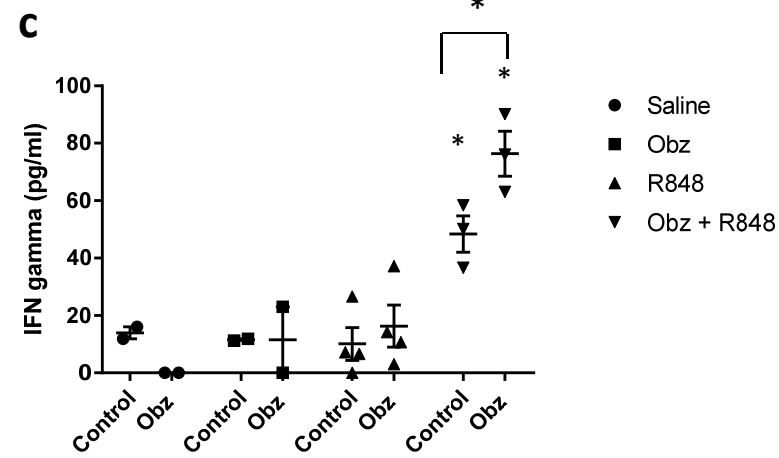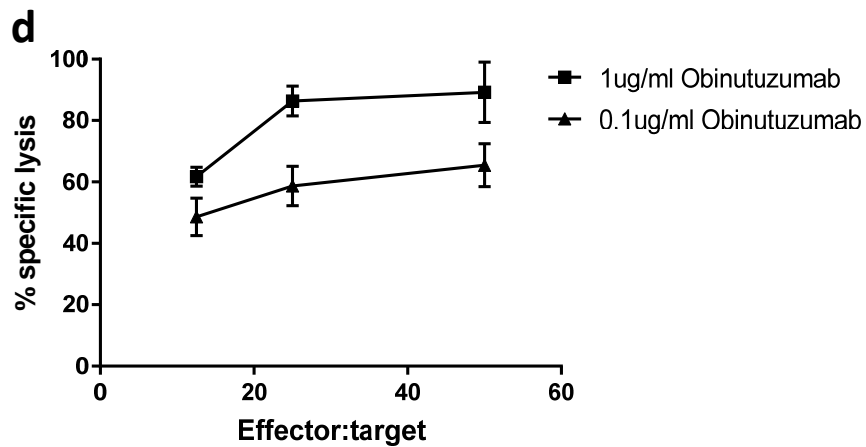

Supplement: Supplementary Figures [file leu2016352x2.pdf]
